# The Long-Term Consumption of Oats in Celiac Disease Patients Is Safe: A Large Cross-Sectional Study

**DOI:** 10.3390/nu9060611

**Published:** 2017-06-15

**Authors:** Katri Aaltonen, Pilvi Laurikka, Heini Huhtala, Markku Mäki, Katri Kaukinen, Kalle Kurppa

**Affiliations:** 1 Center for Child Health Research, Tampere University Hospital, 33521 Tampere, Finland; aaltonen.katri.m@student.uta.fi (K.A.); markku.maki@uta.fi (M.M.); 2 The Faculty of Medicine and Life Sciences, University of Tampere, 33014 Tampere, Finland; katri.kaukinen@uta.fi; 3 Celiac Disease Research Centre, Tampere University Hospital, 33521 Tampere, Finland; laurikka.pilvi.l@student.uta.fi; 4 Faculty of Social Sciences, University of Tampere, 33014 Tampere, Finland; heini.huhtala@uta.fi; 5 Department of Internal Medicine, Tampere University Hospital, 33521 Tampere, Finland

**Keywords:** gluten-free diet, treatment, complications, symptoms, quality of life

## Abstract

A strict gluten-free diet (GFD) can be diversified by non-contaminated oats, but there is a shortage of long-term studies concerning its safety. We compared long-term treatment outcomes and factors associated with the introduction of oats between celiac patients on a GFD with or without oats. Eight hundred sixty-nine previously diagnosed celiac patients were interviewed. The validated Gastrointestinal Symptom Rating Scale (GSRS), Psychological General Well-Being (PGWB), and Short-Form 36 Health Survey (SF-36) questionnaires were used to assess symptoms and quality of life, serological tests were performed, and results of histology were confirmed from patient records. We found the median duration of GFD to be 10 years and 82% using oats. Factors predicting the consumption of oats were diagnosis after the year 2000, advice from a dietitian, detection by screening, and mild clinical presentation. Oat consumers and non-consumers did not differ in dietary adherence (96.5% vs. 97.4%, *p* = 0.746), the prevalence of symptoms (22.9% vs. 22.5%, *p* = 0.931), positivity for endomysial antibodies (8.8% vs. 6.0%, *p* = 0.237), histological recovery after one year (63.1% vs. 60.0%, *p* = 0.773), malignancy (4.8% vs. 3.3%, *p* = 0.420), osteoporosis/osteopenia (9.2% vs. 11.0%, *p* = 0.489), or fractures (26.9% vs. 27.9%, *p* = 0.791). The oat consumers had better SF-36 physical role limitations and general health scores. Based on our results, the long-term consumption of oats in celiac disease patients is safe and may improve quality of life.

## 1. Introduction

By reason of increasing recognition of the diverse clinical presentation of celiac disease, combined with new serological tools for its detection, the condition has emerged as one of the most common nutrition-related diseases [1,2]. The true incidence of the disease seems also to be rising, further emphasizing the considerable public health and economic significance of its optimal management [3,4]. In theory, treatment is simple, as the essential environmental trigger, gluten, is known and can be eliminated by a gluten-free diet (GFD). Notwithstanding its undisputable beneficial effects, a life-long GFD has its disadvantages. It is challenging to maintain and may lead to a restricted and nutritionally suboptimal diet. Further, many GFD products have low fiber and high fat and sugar content, predisposing patients for example to constipation, obesity, and cardiovascular diseases [5,6].

The mainstay of the GFD is the exclusion of dietary wheat, barley, and rye, while the consumption of oats remains controversial. Oats have different storage protein composition than the three other cereals, and the short-term safety of oats in celiac disease patients was proposed as far back as the 1990s [7] and further supported by subsequent clinical trials [8,9,10]. In some studies, however, oats were found to cause symptoms or even occasional small-intestinal damage [11,12] or to trigger immunological responses in experimental models [13,14]. The possibility of using oats would have a major health impact, as it is nutritious and a good source of fiber, which may reduce constipation and the risk of type 2 diabetes [8]. Oats may also lower harmful cholesterol levels, enhance immune defense, and protect against heart disease and cancer [15,16,17]. Finally, oats could diversify the GFD and reduce individual fat and sugar intake. The main limitation in the current evidence of the consumption of oats in celiac disease patients is the scarcity of long-term studies [8,18]. More information would be needed, in particular regarding histological and serological healing and quality of life.

In Finland, oats has traditionally been a major ingredient in the daily diet, and in purified form it was accepted more than 15 years ago and widely used among celiac disease patients [19]. This provided an excellent opportunity to compare long-term treatment outcomes between large and well-defined cohorts of patients on a GFD with or without oats. Additionally, we investigated factors associated with the introduction of oats as a part of the GFD at the time of the celiac disease diagnosis.

## 2. Materials and Methods

### 2.1. Patients and Study Design

Previously diagnosed biopsy-proven celiac disease patients who had been on a GFD for variable periods of time were recruited by a nationwide search via newspaper advertisements and with the help of national and local celiac disease societies. The original diagnosis could have been made at any age, but in the present study only patients currently over 16 years of age were included. Exclusion criteria were unconfirmed diagnosis and markedly lacking medical information either before or at the time of the diagnosis. After entering the study, all voluntary participants were interviewed with structured questions by an experienced physician or study nurse. The interviewers systematically established a variety of celiac disease-related clinical and demographic data as defined below in detail. Further, the participants filled structured gastrointestinal symptom and quality of life questionnaires, and laboratory samples were drawn for further serological analyses. Besides personal interviews, the medical records of each patient were scanned in order to confirm the celiac disease diagnosis and clinical data, and to further explore all relevant histological and serological findings and laboratory values. After data collection, the participants were divided into two groups based on the consumption or non-consumption of oats in their GFD, and all study variables were compared between these groups. 

The Ethical Committee of Pirkanmaa Hospital District approved the study design, patient recruitment, and data collection. Written informed consent was obtained from all enrolled participants.

### 2.2. Clinical Information

Demographic data, celiac disease in the family, time, and site (primary care, secondary care or tertiary care, private sector) of the diagnosis, clinical presentation (e.g., gastrointestinal symptoms, extraintestinal symptoms, and detected by screening), previous and current smoking, and the presence of celiac disease-associated (e.g., dermatitis herpetiformis, type 1 diabetes, and autoimmune thyroidal disease) or other chronic diseases and malignancies were established. In addition, the prevalence of osteoporosis, osteopenia, and any fractures was explored. The durations of symptoms before diagnosis (<1 year, 1–5 years, >5 years) and their severity (mild, moderate, severe) both at diagnosis and at present were recorded. The year of the diagnosis was further sub-classified as before 1990, between 1990 and 1999, and after 2000. The interviewers also inquired whether participants had regular follow-up by health care.

### 2.3. Serology and Histology

Current serum IgA-class endomysial (EmA) and transglutaminase 2 (TG2ab) antibody values were measured in all participants upon study entry. EmA was assessed by indirect immunofluorescence using a human umbilical cord as substrate. Titers of 1:≥5 were considered positive and further diluted up to 1:4000 until negative. TG2ab were assessed by commercial enzyme-linked immunosorbent assay (QUANTA Lite h-tTG IgA, INOVA Diagnostics, San Diego, CA, USA), and values >40 U/L were considered positive. The corresponding IgG-class EmA and TG2ab antibodies were measured in patients with selective IgA deficiency. Since normalization of the antibodies on a GFD may take some time [8,19,20], subjects dieting for less than two years were excluded from the serological follow-up analyses.

Results of diagnostic and follow-up biopsies after one year on GFD were collected from the hospital pathology reports. In our clinical routine, several small-bowel mucosal biopsies are taken from each patient both upon celiac disease suspicion and during the follow-up endoscopy. The histological samples are then forwarded to the pathology department, where well-orientated specimens are carefully evaluated according to our standard operating procedures [21]. Severity of mucosal damage is further categorized as partial (PVA), subtotal (SVA), or total (TVA) villous atrophy, these corresponding roughly to Marsh–Oberhüber grades IIIa, IIIb, and IIIc.

### 2.4. Questionnaires

Self-perceived gastrointestinal symptoms were investigated with a validated Gastrointestinal Symptom Rating Scale (GSRS) questionnaire. The survey consists of 15 separate queries, which can be divided into five sub-domains: indigestion, diarrhea, constipation, abdominal pain, and reflux. Each sub-score is calculated as the average of three relevant items and the total score as the average of all 15 items. Answers are scored using a seven-grade Likert scale (possible points from 1 to 7) with higher scores denoting more severe gastrointestinal symptoms [22].

The Short-Form 36 Health Survey (SF-36) was used to assess quality of life and general health [23,24]. It comprises 36 items representing eight different sub-sections: physical functioning, physical role limitations, emotional role limitations, vitality, mental health, social functioning, bodily pain, and general health. Each item is scored from 0 to 100, and the items in the same section are averaged together to form the eight separate sub-dimensions. Higher scores indicate better health and social functioning.

The Psychological General Well-Being (PGWB) questionnaire is another widely used measure of quality of life and general well-being [25]. PGWB consists of 22 questions representing six different sub-domains as follows: anxiety, depression, well-being, self-control, general health, and vitality. The items use a six-grade scale (points from 1 to 6) and the scores are added together in each different sub-domain and as a total score that can range from 22 to 132 points. Higher scores indicate better health-related quality of life and well-being [26].

### 2.5. Gluten-Free Diet

Duration and strictness of the GFD were asked from all participants. Self-reported dietary adherence was further classified as strict GFD (no lapses), occasional lapses (lapses less than once a month), and no GFD (more common lapses). In addition, the source of dietary advice at the time of diagnosis was established and categorized as no advice, dietitian, or other (e.g., physician or nurse). Finally, regular consumption of oats as part of the GFD was asked about and classified as either use or no use. The GFD label may be used for uncontaminated oats products that contain gluten less than 20 parts per million.

### 2.6. Statistical Analysis

Categorical variables are presented as percentages and continuous variables as medians with ranges or with quartiles as appropriate. Categorical variables were compared using cross-tabulation with a chi-square test. To compare medians between the study groups, the non-parametric Mann–Whitney U test was used. All statistical analyses were made using SPSS version 23. *p*-values < 0.05 were considered statistically significant. Age and sex were considered as possible confounding factors in each analysis.

## 3. Results

### 3.1. Baseline Data and Factors Predicting Oat Consumption

Altogether 869 individuals (median age 53 years, females 75.5%) fulfilled the study criteria and were enrolled. Of these, 715 (82%) consumed oats as part of their GFD. At the time of celiac disease diagnosis 4.4% of the participants were under 16 years of age. Oat-consumers were a few years older at diagnosis, while there was no difference in gender distribution (Table 1). Factors predicting oat-consumption in the GFD were celiac disease diagnosis after the year 2000, detection of the disease by screening, mild clinical presentation at diagnosis, and dietary advice given by a dietitian. The consumption of oats was not dependent on family history of celiac disease, site of diagnosis, duration of symptoms before diagnosis, or severity of small-bowel mucosal damage (Table 1).

### 3.2. Follow-Up Results

There were no significant differences in current ages between the two study groups, but those consuming oats had on average been a shorter time on a GFD before enrolment (Table 2). However, patients in both groups had been on a GFD approximately a median of 10 years (Table 2). They reported excellent and comparable dietary adherence, and there were also no significant differences between the groups in current self-reported symptoms, results of follow-up biopsy or prevalence of celiac disease autoantibody positivity (Table 2). In addition, the median TG2ab values were at the same level (oats 12.0 U/L vs. no oats 10.0 U/L, *p* = 0.077).

The study groups did not differ in the prevalence of osteoporosis, fractures, or malignancies, but subjects on the oat-containing GFD were less often current smokers (Table 2). Further, they were more often completely free of other chronic diseases (16.9% vs. 10.4%, *p*-value = 0.044). In more detailed analysis, however, no significant differences between the groups were found in the prevalence of any specific celiac disease-associated condition (e.g., type 1 diabetes or autoimmune thyroidal disease) or other chronic disease when categorized into major disease groups (metabolic, endocrinological, hematologic, immunologic, ophthalmologic, otolaryngological, gastroenterological, psychiatric, respiratory, locomotor, neurological, gynecological, urologic, and cardiovascular disorders) (data not shown). Furthermore, there was no significant difference between oat consumers and non-consumers in attendance for regular follow-up by health care (Table 2).

In line with the current self-estimated overall symptoms, the groups showed no difference in GSRS total or any sub-dimension scores (Table 3). There was no difference in health-related quality of life when measured by PGWB total and sub-scores, but in SF-36 oat-consumers yielded better scores on physical role limitations and general health (Table 3).

## 4. Discussion

We demonstrated that celiac disease patients consuming oats as part of a longstanding GFD did not differ in symptoms and celiac serology, and had similar or even somewhat better quality of life from those not consuming oats. Further, there was no difference between the groups in small-bowel mucosal damage in control biopsy after one year on a GFD. These findings are in line with most previous short-term studies showing no harm from oat consumption in celiac disease patients [7,9,10], and further strongly support the long-term safety of oats.

One of our aims was to explore factors associated with the introduction of oats as part of the GFD, an issue regarding which there are no previous scientific data. We found oats to be significantly more widely consumed among patients diagnosed after the year 2000 than by those diagnosed earlier. This might be partly a result of physicians’ increased acceptance of oats in the celiac diet. Patients diagnosed by screening and with less severe symptoms were also more likely to consume oats, possibly since they and their physicians are less hesitant to try oats in cases of mild clinical presentation. This is very likely further attributed to the increasing consumption of oats over time, as the screening of celiac disease has also increased during the 2000s [27]. Interestingly, patients who visited dietitians consumed oats more often than those receiving dietary advice from other health care professionals. Dietitians generally have a slightly different perspective on chronic diseases than clinicians [28], and in celiac disease patients they may focus more on the nutritional benefits of oats and recommend it if not specifically forbidden by the responsible physician.

Of note regarding issues not associated with the introduction of oats was the level of health care at which the diagnosis was made. This might not necessarily have been pertinent, as it has been reported that the treatment of chronic diseases differs significantly between general practitioners and specialists [29]. The more uniform results in Finland might be due to the widely used nationwide treatment guidelines for celiac disease [30] and the increasing transfer of the diagnostics from tertiary centers to primary care [27]. We believe that the constantly rising number of celiac patients makes such a decentralization necessary, and there should not be major differences in implementation of the GFD between different levels of health care.

One main finding among long-term outcomes was the absence of any difference between oats and no-oats groups in either self-reported overall symptoms or those measured by validated questionnaire. This is in line with most previous short-term studies [7,9,10,31] and our recent smaller follow-up study [8], in which oats did not increase symptoms on a GFD. However, in our earlier randomized trial oat-consumers reported more diarrhea than those without oats [11], and in a 12-week challenge study from Norway some celiac patients experienced abdominal discomfort and bloating when starting oats [12]. However, since any rapid change in the amount of dietary fiber can cause gastrointestinal symptoms even in non-celiacs [32], the reaction to fiber-rich oats might be only a matter of nonspecific adaptation rather than true immunological activation. In fact, also in the two aforementioned studies [11,12], most patients with initial symptoms later tolerated oats as a part of their GFD. Oats may thus cause symptoms in a small group of celiac patients, but they are usually mild and avoidable by a gradual increase in daily consumption.

Another important result here was the equal self-perceived quality of life in the oats and no-oats groups as measured by validated PGWB and SF-36 questionnaires. In fact, oat-consumers had even somewhat fewer physical role limitations and better general health when measured by the SF-36. Similarly, oat consumption was not associated with decreased quality of life in the above-mentioned randomized trial from our group [11], and in another study celiac patients reported oat consumption as making the GFD easier to maintain by diversifying the diet nutritionally, lowering costs and improving taste [33]. Interestingly, in the current study, we also found oat-consumers to smoke less. This indicates in general a healthier lifestyle, which apparently helps maintain good health and quality of life.

The consumption of oats also did not predispose to a higher risk of celiac antibody positivity or histological damage on a GFD. This is especially important given that the long-term complications of celiac disease are considered to be a consequence of an ongoing intestinal lesion, whose severity the antibody levels also reflect [34]. The excellent morphological mucosal recovery with oats is in line with the findings in our randomized study [11] and more recent studies [8,35,36]. In the first [11] and last [36] of these studies, oat consumers evinced a slightly higher density of duodenal intraepithelial lymphocytes (IELs). The increase was, however, seen mostly in so-called γδ+ IELs, of which eventual significance is unclear and not necessary pathologic. Moreover, the increased levels of IELs had no effect whatsoever on the other measured outcomes and was not seen in the other two studies [8,35]. The fact that the histological damage was not fully healed at the one year control biopsy in up to 40% of the patients in both oats and no oats groups here does not reflect poor dietary adherence, but instead is in line with previous studies showing that, despite a strict GFD, the villous recovery often takes a considerably longer time to recover [36]. Our results are supported by studies from other groups also showing no effect of oats in recovery of the villous architecture [18,37,38,39,40]. Nevertheless, one patient in the above-mentioned Norwegian study [12] developed villous atrophy while using purified oats, and in experimental models of celiac disease, certain oat cultivars have triggered immunological responses [27]. There are also reports of altered epithelial function and avenin-specific T-cell stimulation in a part of patients on a GFD with oats [39,41]. Although these issues need further clarification, true intolerance to oats would appear to be very rare in clinical practice. The safety of oats in the long term was further supported by the equal incidence of malignancies, osteoporosis, and fractures between our study groups. We would also emphasize that, although oats are widely consumed among Finnish patients (here 82%), treatment results are very good and refractory celiac disease is exceptionally rare [42].

Our main strengths were the large study groups with and without oats, the long follow-up time on a GFD, and the use of validated questionnaires for symptoms and quality of life. We also succeeded in collecting a wide variety of clinically relevant follow-up data. One limitation, on the other hand, was that reasons behind the non-consumption of oats were not investigated, and it is possible that in some cases it was initially tried but later omitted due to clinical symptoms [33]. We also had no data as to the exact individual amounts or cultivars of oats consumed, but this reflects the real life setting in which the daily consumption varies substantially both between and within individuals. The mean intake of oats in Finland is approximately 18 g per capita per day [43], and in our previous study [8], the patients consumed 20 g oats per day. Thus, we can assume that the participants were consuming approximately the same amount of oats as the population in general. Another factor we could not control here was that, earlier, the patients might have consumed the so-called naturally gluten-free products of which gluten content was not certified. We also had no data as to the exact individual amounts of oats consumed, but this reflects the real life setting in which the daily consumption varies substantially both between and within individuals. The fact that a part of the participants were members of the celiac society might have caused a selection bias. It is also good to remember that, in Finland, products with purified oats are widely available and strictly regulated [19], and caution is thus warranted before generalizing our results to countries with less experience with such groceries.

To conclude, we provided strong evidence that the consumption of oats as part of the GFD is safe also in the long term in the great majority of celiac disease patients. Considering the various health benefits related to the regular consumption of oats, we encourage physicians to recommend it with a low threshold. It is important to ensure the purity and high quality of oat-containing GFD products [44], and, as always in celiac disease patients, careful monitoring for an adequate response to dietary treatment is mandatory.

## Figures and Tables

**Table 1:** Clinical and histological characteristics and presence of dietary advice at diagnosis in 869 celiac disease patients currently on a gluten-free diet with or without oats.

	Oats *n* = 715	No Oats *n* = 154	
	%	%	*p*-Value
Age at diagnosis, median (range), years	43 (1–81)	41 (1–79)	**0.048**
Females	75.9	73.4	0.502
Celiac disease in the family	66.9	66.0	0.824
Site of diagnosis			0.789
Primary care	14.4	12.3	
Secondary care or tertiary care	72.7	74.0	
Private sector	12.9	13.6	
Year of diagnosis			**<0.001**
<1990	16.4	32.5	
1990–1999	33.3	31.2	
2000–	50.3	36.4	
Clinical presentation at diagnosis			**0.004**
Gastrointestinal symptoms ^1^	56.6	65.6	
Extraintestinal symptoms ^2^	28.1	29.2	
Screen-detected in at-risk groups ^3^	15.2	5.2	
Severity of symptoms before diagnosis ^4^			**0.006**
No or mild	37.2	23.7	
Moderate	12.6	9.6	
Severe	50.2	66.7	
Duration of symptoms before diagnosis			0.186
<1 year	22.2	24.3	
1–5 years	35.8	27.8	
>5 years	42.0	47.9	
Diagnostic histology			0.726
Total villous atrophy	26.4	24.0	
Subtotal villous atrophy	37.6	41.3	
Partial villous atrophy	36.0	34.7	
Dietary advice at diagnosis			**0.006**
No advice	19.7	27.2	
Dietitian	69.3	55.8	
Physician/nurse/other	11.0	17.0	

^1^ E.g., abdominal pain, constipation, diarrhea, malabsorption. ^2^ E.g., arthritis, dental enamel defects, infertility, neurologic symptoms, osteoporosis. ^3^ E.g., relatives of the patients and subjects with type 1 diabetes mellitus or autoimmune thyroidal disease. Data were available in >90% of the subjects in each category except in ^4^ 74%.

**Table 2:** Age at the current study and a variety of follow-up data in 869 celiac disease patients currently on a gluten-free diet (GFD) with or without purified oats.

	Oats *n* = 715	No Oats *n* = 154	
	%	%	*p*-Value
Age at present, median (range), years	53 (17–89)	55 (21–85)	0.716
Time on GFD, median (range), years	9 (1–47)	13 (1–53)	**<0.001**
Current self-reported dietary adherence			0.746
Strict GFD	96.5	97.4	
Occasional lapses	3.2	2.6	
No GFD	0.3	0.0	
Current self-reported symptoms			0.931
No	75.5	75.5	
Mild or moderate	22.9	22.5	
Serious	1.6	2.0	
Follow-up histology on a GFD ^1^			0.773
Healed mucosa	63.1	60.0	
Inflammation/partial villous atrophy	33.5	35.3	
Subtotal/total villous atrophy	3.4	4.7	
Follow-up serology on a GFD ^2^			
Positive EmA	8.8	6.0	0.273
Positive TG2ab	12.2	10.1	0.471
Any malignancy	4.8	3.3	0.420
Osteoporosis or osteopenia	9.2	11.0	0.489
Any fracture	26.9	27.9	0.791
Current smoking	8.2	14.9	**0.009**
Regular follow-up by the health care	29.0	28.7	0.926

^1^ Follow-up biopsy was taken after a median of one year (range: 1–25 years) in both groups. ^2^ Patients with a GFD less than two years were excluded from the analysis. EmA: Endomysial antibodies; TG2ab: Transglutaminase 2 antibodies. Data were available in >90% of the subjects in each variable except in follow-up histology 54%.

**Table 3:** Gastrointestinal Symptom Rating Scale (GSRS), Short-Form (36) Health Survey (SF-36), and Psychological General Well-Being (PGWB) questionnaire scores in 590 celiac disease patients currently on a gluten-free diet with or without oats.

	Oats *n* = 484		No Oats *n* = 106		
	Median	Quartiles	Median	Quartiles	*p*-Value
GSRS scores ^1^					
Total	1.9	1.5–2.5	2.0	1.5–2.7	0.460
Indigestion	2.3	1.8–3.3	2.5	1.7–3.3	0.864
Diarrhea	1.3	1.9–2.3	1.7	1.0–2.3	0.164
Constipation	1.7	1.0–2.7	2.0	1.0–2.7	0.318
Abdominal pain	2.0	1.3–2.3	2.0	1.3–2.7	0.506
Reflux	1.5	1.0–2.0	1.5	1.0–2.5	0.329
SF-36 scores ^2^					
Physical Functioning	95	80–100	90	69–100	0.081
Role limitations, physical	100	50–100	75	25–100	**0.020**
Role limitations, emotional	100	67–100	100	67–100	0.802
Vitality	70	55–85	70	55–85	0.808
Mental health	80	72–88	84	68–92	0.701
Social functioning	88	75–100	88	75–100	0.470
Bodily pain	78	58–90	68	49–90	0.532
General health	65	50–80	60	40–75	**0.048**
PGWB sub-scores ^3^					
Total	106	94–115	104	95–116	0.526
Anxiety	25	21–27	25	22–27	0.658
Depression	17	15–18	16	15–18	0.215
Well-being	18	15–20	17	14–20	0.628
Self-control	16	14–17	16	14–17	0.952
General health	13	11–15	13	10–15	0.128
Vitality	18	16–20	18	16–21	0.515

Higher scores denote either ^1^ more severe symptoms, ^2^ better health and social functioning, or ^3^ better health-related quality of life.
